# Plasmodium Perforin-Like Protein Pores on the Host Cell Membrane Contribute in Its Multistage Growth and Erythrocyte Senescence

**DOI:** 10.3389/fcimb.2020.00121

**Published:** 2020-03-24

**Authors:** Swati Garg, Abhishek Shivappagowdar, Rahul S. Hada, Rajagopal Ayana, Chandramohan Bathula, Subhabrata Sen, Inderjeet Kalia, Soumya Pati, Agam P. Singh, Shailja Singh

**Affiliations:** ^1^Department of Life Science, School of Natural Sciences, Shiv Nadar University, Greater Noida, India; ^2^Laboratory of Neuroplasticity and Neuroproteomics, Department of Biology, KU Leuven, Leuven, Belgium; ^3^Department of Chemistry, School of Natural Sciences, Shiv Nadar University, Greater Noida, India; ^4^Infectious Diseases Laboratory, National Institute of Immunology, New Delhi, India; ^5^Special Centre for Molecular Medicine, Jawaharlal Nehru University, New Delhi, India

**Keywords:** perforin like proteins, malaria, erythrocyte, anemia, invasion, egress, atomic force microscopy, Raman spectroscopy

## Abstract

The pore forming *Plasmodium* Perforin Like Proteins (PPLP), expressed in all stages of the parasite life cycle are critical for completion of the parasite life cycle. The high sequence similarity in the central Membrane Attack Complex/ Perforin (MACPF) domain among PLPs and their distinct functional overlaps define them as lucrative target for developing multi-stage antimalarial therapeutics. Herein, we evaluated the mechanism of Pan-active MACPF Domain (PMD), a centrally located and highly conserved region of PPLPs, and deciphered the inhibitory potential of specifically designed PMD inhibitors. The *E. coli* expressed rPMD interacts with erythrocyte membrane and form pores of ~10.5 nm height and ~24.3 nm diameter leading to hemoglobin release and dextran uptake. The treatment with PMD induced erythrocytes senescence which can be hypothesized to account for the physiological effect of disseminated PLPs in loss of circulating erythrocytes inducing malaria anemia. The anti-PMD inhibitors effectively blocked intraerythrocytic growth by suppressing invasion and egress processes and protected erythrocytes against rPMD induced senescence. Moreover, these inhibitors also blocked the hepatic stage and transmission stage parasite development suggesting multi-stage, transmission-blocking potential of these inhibitors. Concievably, our study has introduced a novel set of anti-PMD inhibitors with pan-inhibitory activity against all the PPLPs members which can be developed into potent cross-stage antimalarial therapeutics along with erythrocyte senescence protective potential to occlude PPLPs mediated anemia in severe malaria.

## Introduction

Malaria remains a serious global health challenge and major roadblock for the economic growth of the poor and developing economies. The rapid emergence of drug-resistant malaria parasites has exceeded the rate at which anti-malarial therapies are presently being introduced. Though currently available antimalarial therapies target blood-stage to reduce the disease burden, the next-generation therapeutics demands development of drugs with potent cross-stage protection, for complete prevention (WHO, 2018). Therefore, an efficient treatment regimen needs to be both curative and transmission-blocking. In lieu of the same, molecular players performing multiple roles across the life cycle of the malaria parasite would thus serve as ideal targets for developing pan-active therapeutic interventions. In this regard, *Plasmodium* Perforin like proteins (PPLPs) are excellent candidates in this regard and need to be further characterized.

Perforin like proteins (PLPs) are the eukaryotic pore forming proteins conserved across the apicomplexan parasites, and are the crucial players in the biology of malaria parasite across all the stages of *Plasmodium* life cycle (Tavares et al., 2014; Alaganan et al., 2017). The genome of *Plasmodium* spp. encodes for five PPLPs (PPLP1-5) that work in different combinations at different stages of the parasite life cycle, and are indispensable for the parasite growth and survival (Kadota et al., 2004; Ishino et al., 2005; Ecker et al., 2007; Deligianni et al., 2013; Wirth et al., 2014, 2015; Yang et al., 2017). In the liver stage, PPLP1 has a distinct role in the successful establishment of hepatocyte infection (Ishino et al., 2005; Yang et al., 2017). By HA tagging of the PPLP1 locus followed by immunoblotting with HA antibody, Yang et al. could not detect its expression in the blood stage. However, other reports confirm the expression of PPLP1 and PPLP2 in blood-stage schizonts and merozoites using LC-MS/MS (Lasonder et al., 2002; Garg et al., 2013). PPLP1 and PPLP2 are involved in the permeabilization of the host erythrocyte membrane during the egress of malarial parasites (Garg et al., 2013). In the gametocytes, PPLP2 is responsible for the egress of activated gametocytes from the host erythrocytes (Deligianni et al., 2013; Wirth et al., 2014). PPLP3, PPLP4, and PPLP5 are expressed in ookinete and are involved in mosquito midgut traversal to form oocysts (Kadota et al., 2004; Ecker et al., 2007; Wirth et al., 2015). Despite the importance that PPLPs have in the parasite life cycle, no chemotherapeutic interventions have been developed against them. Although single knockout suggests that individually they are not essential for the blood stage, functional knockout of both PfPLP1 and PfPLP2 can better reveal their role. Since the double knockout is a challenging task for malaria parasites, inhibitors can be mimic of functional knockout of PfPLPs. A few reported inhibitors for eukaryotic pore-forming proteins identified till date, mostly exert their effects indirectly through inhibition of protein processing, storage, or secretion from organelles rather than directly inhibiting perforin's function in the target cell (Kataoka et al., 1996a,b). Recently, a few small molecule inhibitors were identified from a high throughput screening which inhibited mouse perforin at sub-micromolar doses (Lena et al., 2008; Spicer et al., 2013). These studies proved motivational for the development of anti-PPLP molecules, which required identification and functional characterization of a common motif of PPLPs that can serve as the universal target for chemotherapeutics.

PPLPs have an N-terminal signal sequence, a central MACPF (membrane attack complex/ perforin) domain, and a C-terminal β-sheet rich domain (Garg et al., 2015). The central MACPF domain is the functional unit of PPLPs containing the characteristic signature motif of eukaryotic pore-forming proteins and two transmembrane helical domains (CH1 and CH2) that exhibit the typical arrangement of alternate hydrophilic and hydrophobic residues and is involved in membrane insertion (Hadders et al., 2007; Pipkin and Lieberman, 2007; Rosado et al., 2007). The C-terminal domain (CTD) is rich in the β-sheet and is involved in membrane binding similar to eukaryotic pore-forming proteins (Voskoboinik et al., 2005). PPLP molecules are initially secreted as monomers that bind to the target cell membrane and oligomerize on its surface to form functional, transmembrane pores (Pipkin and Lieberman, 2007; Baran et al., 2009). The structure of PPLPs remains unsolved, however, recently the crystal structure of a closely related PLP of another apicomplexan parasite *Toxoplasma gondii*, TgPLP1 was deciphered (Gilbert et al., 2018). The structure of TgPLP1 is similar to the reported eukaryotic pore-forming proteins, but, bulkier than others due to the presence of extra helices that may play roles in pore formation (Gilbert et al., 2018). The structure of PPLPs can now be predicted using TgPLP1 as a template for designing anti-malarial chemotherapeutics.

In this study, using various biochemical, biophysical, and pharmacological evidences, we characterized the pore forming activity of PLPs on erythrocytes. Further, the specifically designed inhibitors could restrict this pore formation, impede the exit/entry of the parasites and also could exert multiple-stage inhibition and rescue the uninfected erythrocytes from death. Together, we highlight the mechanism of pore formation by PPLPs and evaluate their potential for the development of pan-active inhibitors to provide both symptomatic and transmission-blocking cure for malaria.

## Materials and Methods

### Expression and Activity of Recombinant Pan-MACPF Domain 1 (rPMD1) and Recombinant Pan-MACPF Domain 2 (rPMD1)

Codon optimized gene encoding for rPMD1 (Figure S1A) and rPMD2 domain (Figure S1B) were subcloned into bacterial expression vector PET28a (+) and protein expression in *E.coli* cells was induced with 1 mM isopropyl-β-D-thiogalactoside (IPTG). His-tagged rPMD1 was purified from inclusion bodies while his-tagged rPMD2 was purified from the soluble fraction using Ni-NTA chromatography. The gene encoding for rPLP2 C-terminal domain was subcloned into bacterial expression vector pQE 30. The protein expression in *E. coli* cells was induced with 1 mM isopropyl-β-D-thiogalactoside (IPTG) and the his-tagged protein was purified from the soluble fraction using Ni-NTA chromatography. The concentration of purified proteins was measured using the BCA estimation kit (Pierce, USA).

### Erythrocyte Lysis and Permeabilization Assays

5 × 10^6^ human erythrocytes were incubated with rPMD1 or rPMD2 in lysis buffer for 1 h at 37°C as described previously (Garg et al., 2013). The release of hemoglobin into the supernatant was estimated by measuring absorbance at 405 nm. RBCs incubated in lysis buffer alone or rPPLP2-C-ter protein were taken as the negative control. Maximum hemoglobin release is lysis of 5 × 10^6^ human RBCs in water which is considered as 100% lysis. For the permeabilization assays, lysis was performed in the presence of Rhodamine-phalloidin and 10 kDa FITC-dextran.

### Binding and Oligomerization Assays

Binding of rPMD1and rPMD2 to human erythrocytes was performed as described previously (Gaur et al., 2007). Briefly, erythrocytes were incubated with rPMD1 or rPMD2 for 1 h at 4°C and bound proteins were eluted using 1.5 M NaCl and detected by Western blotting. For the oligomerization assay, rPMD1 or rPMD2 was incubated with human erythrocytes for 1 h at 37°C and centrifuged. The oligomerized protein was detected in erythrocyte pellets using Western Blot analysis (Garg et al., 2013).

### Modified Cellular Thermal Shift Assay (CETSA)

Interaction between the PMIs with rPMDs was tested using CETSA as described previously (Jafari et al., 2014). Briefly, the rPMDs alone or in combination with the compounds were treated at 4, 60, and 80°C and cooled down followed by centrifugation. The supernatant was analyzed by SDS-PAGE. The band intensities for each of the protein lane was determined using Image J software (NIH, USA) and plotted considering the band intensity of untreated 4°C erythrocytes as maximum (100%).

### *In vitro* Culture of *P. falciparum*

Laboratory strain of *P. falciparum*, 3D7 was cultured in RPMI 1640 (Invitrogen, USA) supplemented with 27.2 mg/L hypoxanthine (Sigma Aldrich, USA) and 0.5% Albumax I (Invitrogen, USA) using O+ erythrocytes in mixed gas environment (5% O_2_, 5% CO_2_, and 90% N_2_) as described previously (Trager and Jensen, 1976). Parasites were synchronized by sorbitol selection of rings and percoll selection of schizonts.

### HepG2 Cytotoxicity Assay

Human liver hepatocellular carcinoma cell line (HepG2 cells) were cultured in Dulbecco's Modified Eagle's Medium as described previously (Ramu et al., 2017). HepG2 cells were seeded at a density of 30,000 cells per well and allowed to grow overnight. Adhered cells were treated PMIs and kept for 48 h. Cytotoxic effect was assessed using MTT (3-[4,5- dimethylthiazol-2-yl]-2,5-diphenyl tetrazolium bromide) assay (Sigma-Aldrich, USA).

### Parasite Growth Inhibition Assay (GIA)

To access the effect of PMIs on parasite growth, synchronized trophozoites were treated with different concentrations of C01 or C02 compounds along with DMSO control at 37°C for one cycle of parasite growth. The smears were prepared after 48 h and stained with Giemsa solution and scored for infection under a light microscope. IC_50_ was calculated using graph pad prism 8.0 (CA, USA). To access the non-specific effect of PMIs on erythrocytes, erythrocytes were treated with different concentrations of C01 or C02 at 37°C for 3 h and washed with incomplete RPMI. Purified schizonts were added to treated erythrocytes at a hematocrit of 2% and parasitemia of 1.0%. Parasites added to untreated erythrocytes were used as a control. The parasite invasion was scored after 8 h by counting the number of infected erythrocytes in Giemsa (Sigma, USA) stained smears under the light microscope.

### Parasite Egress and Invasion Assay

To assess the effect of PMIs on parasite egress, late-stage schizonts [~46 h post-invasion, (hpi)] were diluted to a final hematocrit of 2% and parasitemia of ~10% and treated with different concentrations of C01 and C02 along with DMSO control for 6 h. Subsequently, the erythrocytes were smeared and counted for schizonts as well as rings by staining with Giemsa under the light microscope. Percent Egress was calculated as the fraction of schizonts ruptured in treatment and control during the incubation time as compared with the initial number of schizonts at 0 h, using the formula as described below. Percent Egress was then plotted considering the fraction of schizonts ruptured in control as 100% egress.

$$\begin{array}{l} \text{\%Egress}=100\left[\left(\text{I}-\text{T}\right)/\left(\text{I}-\text{C}\right)\right] \end{array}$$

I, initial no. of schizonts; T, no. of schizonts in treatment; C, no. of schizonts in DMSO control.

For scoring invasion, the number of rings formed per egress of schizont was counted and plotted.

### Ring and Trophozoites Toxicity Assay

To assess the effect of C01 and C02 on the ring stage, early rings (~6 hpi) at a parasitemia of 1% were treated with different concentrations of PMIs along with DMSO control. All the wells were washed following 6 h and replenished with media and harvested after 48 h. The parasite growth was assessed by fluorimeter using SYBR green (Thermo Fisher Scientific, USA) staining. For trophozoites toxicity assay, early trophozoites instead of rings were taken and a similar protocol was performed.

### *In vitro* Growth Inhibition Assay for Liver-Stage Parasites

The diluted PMI solutions were added to 24-well culture plates (final DMSO concentration was 1%) containing human HepG2 cells seeded a day prior to the experiment and 0.5 ml complete Dulbecco modified Eagle medium (DMEM) containing 10% fetal bovine serum (FBS), together with a cocktail of penicillin, streptomycin and amphotericin. Infection was initiated by adding 10,000 *Plasmodium berghei* ANKA sporozoites. Infected cultures were then allowed to grow at 37°C in a 5% CO_2_ atmosphere for 51 h. The culture medium was changed 24 h after infection, and fresh compounds were added at the same concentration as on the previous day, to maintain inhibitor pressure throughout the growth period. At the end of the 51 h incubation period, total RNA was extracted using TrizolTM (Invitrogen, USA) reagent. Reverse transcription from 1 μg RNA was performed using a cDNA synthesis kit (GCC Biotech, India) to obtain cDNA. In a real-time PCR mix (H-eff qPCR mix, GCC Biotech, India) of 20 μl, a cDNA equivalent of 0.1 μg RNA was used. The real-time PCR mix also contained *P. berghei* 18S rRNA specific primers. Real-time PCR was performed using an Eppendorf Mastercycler realplex4, and the copy numbers were calculated, using the known amount of plasmid standard having the amplification target sequence. Parasite growth inhibition was calculated by dividing the 18S rRNA copy number of the experimental group by that of the untreated control group. The fraction obtained was then converted into % inhibition (with respect to untreated as 100%).

### Annexin and Calcium Staining Assays

Erythrocytes were treated with a sublethal concentration of rPMD1 or rPMD2 along with the different concentrations of C01 and C02. The samples were incubated for 48 h at 37°C and 5% CO_2_. After 48 h, the samples were stained with Annexin-FITC (Life Technologies, USA) and Fluo-4 AM (Life Technologies, USA). The erythrocytes were imaged using a Nikon A1R microscope and also quantified using FACS BD Fortessa (Becton & Dickinson, USA) using Cell Quest software by scoring 100,000 cells per sample. Samples were analyzed using FlowJo software (Tree Star Inc, Ashland) by determining the proportion of FL-2 positive cells in comparison to the stained untreated erythrocytes.

### Live-Cell Microscopy and Flow Cytometry for Calcium Influx

The erythrocytes were loaded with Fluo-4 AM (Life Technologies, USA), placed in coverslip bottom petri dishes and observed under a confocal microscope equipped with a temperature-controlled stage (Nikon A1R). These erythrocytes were treated with a sub-lethal concentration of rPMD1 (1 μg) or rPMD2 (50 ng) in the presence or absence of PMIs. DIC and fluorescent images were captured using a 100X, 1.4 numerical aperture lens at 1 frame per second. The recording was started for 30 s and then rPMD2 (rPMD2 along with PMI in case of inhibitor treatment) was added and captured for a total of 10 min at 37°C. The staining was also quantified using BD Fortessa (Becton & Dickinson, USA) by scoring 100,000 cells per sample and analyzed using FlowJo software (Tree Star Inc, Ashland).

### Gametocyte Exflagellation Assay

To initiate *P. falciparum* gametocyte cultures, synchronized asexual blood-stage cultures were grown to a parasitemia of 10–15%, treated with 50 mM *N*-acetyl-D-glucosamine containing medium for 4 days to remove asexual stages and maintained in complete RPMI/HEPES to allow gametocyte development. Stage V gametocytes were incubated with activation medium (100 nM xanthuneric acid (XA), 20% AB+ human serum in RPMI1640/HEPES) in the presence of C01 and C02 at 25°C. Activated gametocytes were spread on glass slides 5 min after activation, fixed with methanol and stained with Giemsa (RAL Diagnostics, France). Around 200–250 gametocytes were scored to determine the percentage of rounded up gametocytes. The number of exflagellation centers was scored in gametocyte cultures by light microscopy at 40X magnification in 30 optical fields 15 min after activation.

### Surface Plasmon Resonance

To determine the C01 and C02 interaction with rPMD11, SPR was performed using Auto-Lab Esprit SPR. rPMD1 (10 μM) was immobilized on the surface of the nickel-charged NTA SPR chip. Interaction analysis was studied by injecting C01, C02 along with rPMD1 over the chip surface, with association and dissociation time of 300 and 150 s, respectively. HEPES buffer was used both as immobilization and binding solutions. The surface of the sensor chip was then regenerated with a 50 mM NaOH solution. Data were fit by using Auto-Lab SPR Kinetic Evaluation software provided with the instrument.

### *In silico* Docking

Using I-Tasser and PHYRE2, domain-based modeling and threading were performed to obtain the 3D structures of *P. falciparum* PLP1-5. Following this, structure refinement was done using ModRefiner. To characterize the catalytic pocket, SiteHound software was used while utilizing the structural motif of the MACPF domain in each of the proteins (PLP1-5). Further, molecular docking of two PMIs (C01 and C02) was done using AutodockTools and Autodock vina with an exhaustiveness cut-off of eight for each run. All the ligands were kept flexible by examining torsions.

### Atomic Force Microscopy

Erythrocytes treated with rPMD or in combination with PMIs were smeared and air-dried on a clean grease-free glass slide and imaged using WITec alpha using NSG30 probes with force constant of 22–100 N/m, the resonant frequency of 240–440 Hz, tip curvature radius of 10 nm (Tips nano) in non-contact mode. Topographic images were obtained at points per line and lines per image of 512 ×512 with the scan rate of 0.5 times/line (Trace) (s). All the AFM images were recorded using the Control Four 4.1 software. The images were 3D processed and analyzed using software Project Four 4.1 software (WITec, Germany).

### Raman Imaging and Analysis

The Raman measurements of all the samples were performed using WITec alpha 300RA combined confocal Raman microscope. The spectrometer was equipped with solid-state diode lasers operating 532 nm and a suitable CCD detector that was cooled to −60°C. A Zeiss Fluor (100X) EC Epiplan-NEOFLUAR objective was used. The spectral resolution was equal to 1cm^−1^. The integration time for a single spectrum varied from 2 to 5 s. Raman measurements and data analysis were performed using software Project Four 4.1 software (WITec, Germany). All Raman spectra presented were after pre-processing (baseline correction, smoothening and background removal) using asymmetric least squares smoothing method.

### Statistical Analysis

For all the experiments, the data are presented as the mean ± standard deviation of the results and the number of biological replicates per experimental variable (n) is given in the figure legends. The data for the half-maximal inhibitory concentration (IC_50_) value and the % growth inhibition activity of compounds were analyzed and calculated using non-linear regression in Graph Pad Prism 8 (CA, USA). A student's *t*-test was performed to calculate the *p-*values, where *p* < 0.05 represents ^*^, *p* < 0.01 represents ^**^, and *p* < 0.005 represents ^***^ significance.

## Results

### The Pan-MACPF Domain of PfPLPs Forms Pores at Physiological Relevance

**Figure 1:**
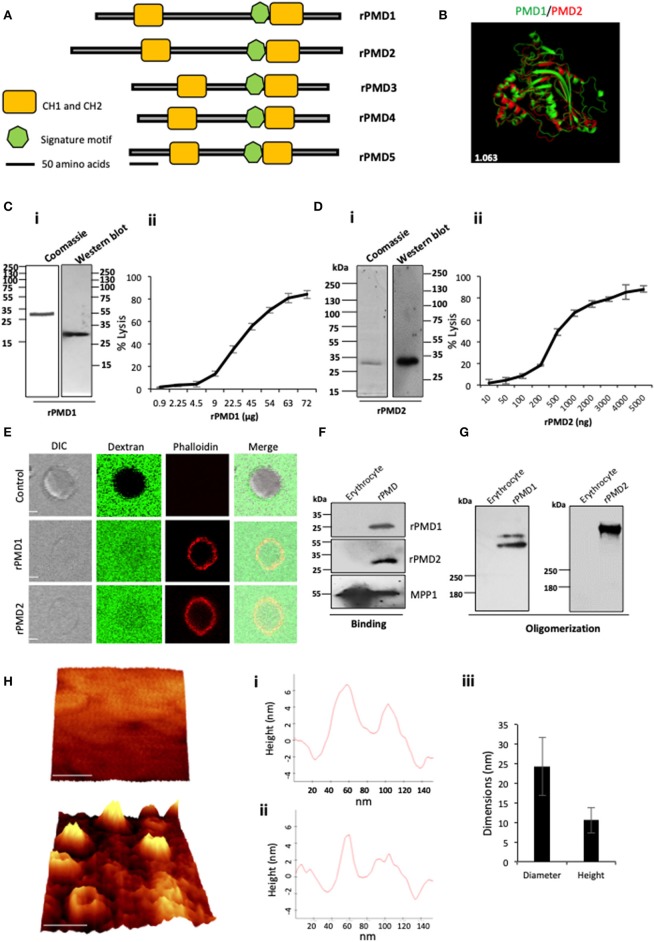
Purification and activity of rPMDs. **(A)** Domain architecture of the MACPF domain of PfPLPs. The signature motif (green box) and two transmembrane helical domains, CH1 and CH2 (yellow boxes) are depicted. Scale bar represents 50 aa. **(B)** Structural superimposition of PMD domain of PfPLP1 and PfPLP2. RMSD value is indicated in white. **(C)** (i) Coomassie and Western blot of affinity-purified rPMD1 probed with the anti-his antibody. (ii) The dose-dependent membranolytic activity of rPMD1. The lysis of human erythrocytes was analyzed in the presence of different concentrations of rPMD1. The graph indicates the percent lysis of human erythrocytes by rPMD1 as compared with 100% hypotonic lysis of human erythrocytes in water (*n* = 3). **(D)** (i) Coomassie and Western blot of affinity-purified rPMD2 probed with the anti-his antibody. (ii) The dose-dependent membranolytic activity of rPMD2. The lysis of human erythrocytes was analyzed in the presence of a different amount of rPMD2. The graph indicates the percent lysis of human erythrocytes by rPMD2 as compared with 100% hypotonic lysis of human erythrocytes in water (*n* = 3). **(E)** Permeabilization activity of rPMD1 and rPMD2. The human erythrocytes were incubated with Phalloidin Alexa 594 and 10 kDa FITC-Dextran in the presence and absence of rPMD1 and rPMD2 and visualized under a confocal microscope. Phalloidin staining was detected in the human erythrocytes treated with rPMD1 and rPMD2 but not in untreated human erythrocytes. **(F)** Western blot analysis of bound rPMD1 and rPMD2 eluted by 1.5 M NaCl. **(G)** Oligomerization of rPMD1 and rPMD2. rPMDs are incubated with human erythrocytes at 37°C for 30 min and analyzed for oligomeric rPMD1 and rPMD2 by Western blotting. **(H)** Visualization of oligomeric pores by AFM. (i) Erythrocytes were treated without (top) or with rPMD2 (bottom) for 30 min at 37°C and visualized under the AFM for pore formation. The image was 3D constructed using project Witec 4.1 software (*n* = 2). Scale bar represents 50 nm. (ii) Line profile of rPMD2 oligomers. Representative images of the height of the oligomer measured along the pore in AFM topographs are depicted. (iii) The average ring diameter and height of rPMD2 treated oligomers protruding from the erythrocytes membrane. Diameter and height were measured for oligomers formed on the erythrocyte membrane. Bars represent an average of 30 oligomers and error bars SD.

The presence of the MACPF domain in PfPLPs is necessary for pore formation. We mapped the minimal, active domain of PfPLPs and named it as pan-MACPF domain (PMD) (Figure S2A). The PMD harbors a signature motif ((Y/W)-X6-(F/Y)GTH(F/Y)-X6-GG) along with two clusters of helices (CH1 and CH2) to form pores in the cell membrane (Figure 1A). Interestingly, the sequence analysis of different PMDs revealed that they are highly conserved and closely related to each other. This stretch of a sequence is also evolutionarily conserved across *Plasmodium* spp. (Figure S3).

To investigate whether the conservation of sequence is also reflected in structural conservation, we *in silico* modeled the structure of PMDs based on the MACPF domain of a closely related apicomplexan parasite *T. gondii*, TgPLP1. Like other MACPF domains, PMDs contain a central β-pleated sheet, surrounded by CH1 and CH2 on either side. In addition, two helical inserts are additionally present in PMDs, similar to TgPLP1, that is absent in all other reported structures of the MACPF domain. Overall, the structural prediction revealed significant conservation of the MACPF domain fold across different PMDs (Figure 1B and Figure S2B).

The expression and purification of the recombinant MACPF domain from the earlier reported mammalian or insect cell system yield insufficient quantities of recombinant protein. To characterize, the pore-forming activity of PMDs *in vitro*, we cloned the codon optimized MACPF domain of PfPLP1 (rPMD1) and PfPLP2 (rPMD2) in pET28a (+) and recombinantly expressed rPMD1 and rPMD2 in *E. coli*. We could successfully purify the active protein from bacteria (Figures 1Ci,Di) and demonstrate the erythrocyte lysis activity of both, rPMD1 and rPMD2, *in vitro* (Figures 1Cii,Dii). Both PMDs lysed erythrocytes in a dose-dependent manner which is due to their pore-forming activity on the erythrocyte membrane. Whereas, the histidine-tagged, C-terminal domain of PPLP2 did not demonstrate any lytic activity toward erythrocytes further confirming that the lytic activity of PPLPs is due to the central MACPF domain only (Figures S4A–C).

To confirm the permeabilization activity of rPMDs, the activity of rPMD1 and rPMD2 were monitored in the presence of rhodamine Phalloidin and 10 kDa FITC-dextran. The rPMD1 and 2 treated erythrocytes displayed phalloidin positivity accompanied by the uptake of 10 kDa FITC-dextran indicating permeabilization of the cell membrane (Figure 1E).

The PFPs binds to lipid bilayer and oligomerizes on it to create pores. To test the binding of rPMDs to the erythrocytes, sublytic concentration of rPMDs was incubated with erythrocytes and their binding was detected by Western blotting. We found that the monomers of rPMD1 and rPMD2 can bind to erythrocytes (Figure 1F and Figure S4D). We further investigated whether the binding of rPMDs leads to their oligomerization. To test this, lytic concentrations of rPMD1 and rPMD2 were incubated with erythrocytes and the ghosts thus formed were evaluated rPMD oligomers by Western Blot analysis. rPMD1 and rPMD2 formed SDS resistant, higher molecular weight oligomers (>250 kDa) suggesting the involvement of more than 8 monomers in the formation of pores (Figure 1G).

Although many studies indicate the pore-forming capacity of PPLPs, this process has not been characterized in detail. Hence, to gain in-depth insights into the characteristics of PPLP pores, we performed high-resolution atomic force microscopy (AFM) of oligomers on the erythrocyte membrane. Erythrocytes serve as the physiological host of PfPLP1 and PfPLP2 *in vivo* and hence we performed studies on red cells rather than model lipid bilayer membranes. AFM topographs of erythrocytes incubated with rPMD2 showed pore-forming oligomers in circular forms (Figure 1H). Height analysis demonstrated that rPMD2 oligomers have a vertical measurement of 10.55 ± 3.22 nm (mean ± SD, *n* = 30) on the erythrocyte surface (Figure 1Hi,ii). The diameter of oligomers was distributed widely between 10 and 40 nm, showing a mean at 24.33 ± 7.42 nm (mean ± SD, *n* = 30). The line profile reveals the height and diameter of a single pore formed by rPMD2 (Figure 1Hiii). Overall, this result confirms that PMD can drill pores in the membrane of erythrocytes.

### Sub Lytic Concentration of PMDs Trigger Premature Senescence of Bystander Erythrocytes

**Figure 2:**
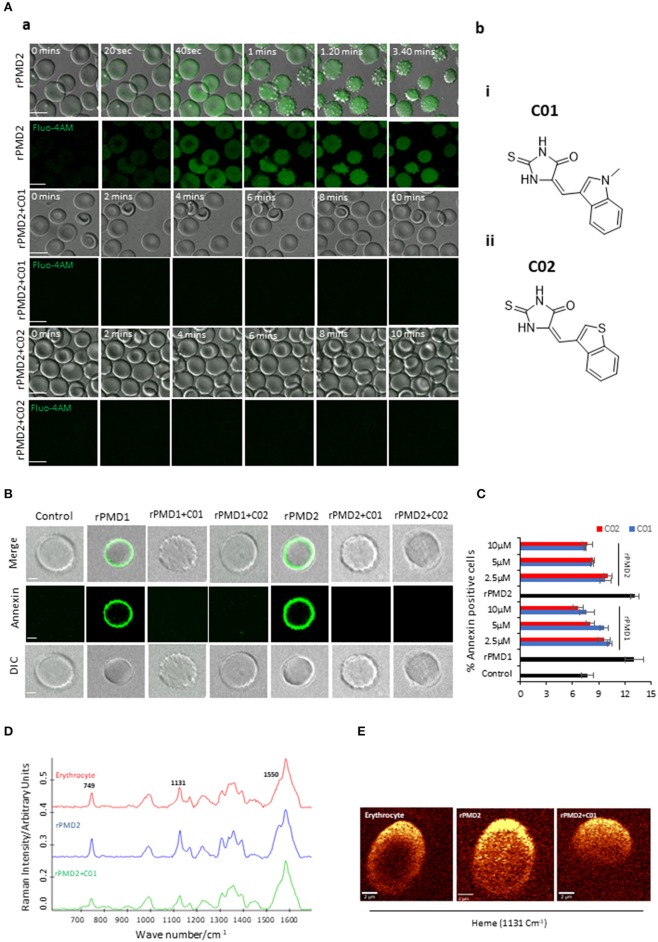
Inhibition of bystander effect mediated by C01 and C02. **(A**-a**)** Time course of rPMD2-induced calcium channel formation. Erythrocytes were loaded with Fluo-4 AM. Sub lytic concentration of rPMD2 (50 ng) was added and the increase in calcium was monitored by confocal microscopy. Selected pairs of DIC and fluorescence images with time elapsed between frames in seconds (Sec) are shown. In the case of PMI treatment, C01 or C02 were added along with rPMD2. **(A-**b**)** (i) The structures depict scaffold of (Z)-5-((1-methyl-1H-indol-3-yl)methylene)-2-thioxoimidazolidin-4-one (C01) and (ii) (Z)-5-(benzo[b]thiophen-3-ylmethylene)-2-thioxoimidazolidin-4-one (C02). **(B)** Phosphatidylserine exposure on the erythrocyte surface. The erythrocytes were treated with rPMD1 or rPMD2 in the presence or absence of PMIs and stained with Annexin V-FITC after 48 h. The stained erythrocytes were visualized under a confocal microscope. **(C)** The annexin positive erythrocytes were quantitated using a flow cytometer. **(D)** Average Raman spectra of the untreated erythrocytes or erythrocytes treated with rPMD2 in the presence or absence of C01 was captured using 532 nm excitation. All the Raman spectra were presented after pre-processing (baseline correction, smoothening and background removal) using asymmetric least squares smoothing method (*n* = 2). **(E)** Raman images of erythrocytes were observed at 1,131 cm^−1^ which demonstrates the distribution of methemoglobin (*n* = 2).

The evolutionary conservation of PPLPs and their importance in disease pathogenesis prompted us to develop novel inhibitors against pan-MACPF domain. Recent reports demonstrate the identification of anti-perforin inhibitors through a high-throughput screen (Miller et al., 2016). Inspired by this, anti-PMD inhibitors (PMI), C01 and C02, were designed with a hypothesis that they can be pan-active against all PPLPs (Figure 2Ab,i,ii).

An earlier report has revealed that secretion of TgPLP1 from one parasite can induce pore formation and egress of bystander parasites (Kafsack et al., 2009). Since PfPLP1 and PfPLP2 are secreted during the egress of merozoites and binds to the erythrocyte membrane (Garg et al., 2013), we thought to analyze the role of secreted PfPLPs on RBC senescence. We incubated the purified late-stage schizonts (~44 h) with uninfected erythrocytes for 10 h at 37°C. To determine the effect of purified rPMDs on the bystander erythrocyte, time-lapse microscopy was performed, mimicking *in vivo* condition. Addition of sublytic concentration of rPMD1 (1 μg) and rPMD2 (50 ng) induced calcium influx in erythrocytes leading to changes in erythrocyte deformability followed by echinocytosis (Figure 2A-a, Movie 1). Initially, to check the non-specific calcium increase, we incubated the erythrocytes with PMIs that did not show any calcium increase (Figure S5A). Moreover, the PMIs alone also could not cause any erythrocyte lysis (Figure S5B). Inhibition of PMD binding to erythrocyte by PMIs restricted the influx of calcium and echinocytosis (Figure 2A-a, Figure S5Ci,ii and Movies 2, 3). The calcium intake was further quantitated by flow cytometry also depicted an increase in intracellular calcium in response to rPMDs (Figure S5C). These results suggest that PfPLPs form smaller pores on erythrocytes at sub lytic concentrations that induce calcium influx but do not lead to lysis of erythrocytes.

Calcium influx also leads to phosphatidylserine (PS) exposure on the surface of erythrocytes that can lead to phagocytosis of cells by macrophages (Callahan et al., 2003; Brown and Neher, 2012). To assess this effect, erythrocytes were treated with sub lytic concentrations of rPMD1 and rPMD2 in the presence and absence of PMIs and stained with Annexin V-FITC after 48 h. The Annexin V-FITC positivity was analyzed by microscopy and flow cytometry (Figures 2B,C and Figure S5D). rPMDs treated erythrocytes in the absence of PMIs were Annexin-positive suggesting induction of PS exposure while the presence of PMIs abrogated their Annexin positivity (Figure 2C). This result indicates that at sub lytic concentrations, PfPLPs can induce delayed erythrocyte sequestration.

Oxidation of hemoglobin, leading to the formation of methemoglobin, is a marker for erythrocyte senescence (Umbreit, 2007). The methemoglobin carries the oxidized form of the heme (Fe^3+^) that leads to changes in its porphyrin ring (Umbreit, 2007). To detect these changes in heme, we used Raman spectroscopy that is a non-invasive and label-free technique for the detection of metabolic changes at the single-cell level (Barkur et al., 2018). When 532 nm is applied to rPMD2 treated erythrocytes, *v*_15_ gains intensity (pyrrole gains intensity) which is co-related well with the increase in intensity at band 749 cm^−1^. A similar increase of band intensity was observed for the band located at 1,131 cm^−1^ that suggests the asymmetrical pyrrole half-ring stretching vibration (Figure 2D). The 1,550 cm^−1^ demonstrates the stretching mode *v* (CbCb) (Dybas et al., 2018). The untreated and PMI treated erythrocytes demonstrated similar peak profiles (Figure 2D). Further, Raman imaging of 1,131 cm^−1^ also confirmed an increase in methemoglobin concentration within erythrocytes (Figure 2E). These results suggest the formation of methemoglobin following rPMD2 treatment that could lead to erythrocyte senescence.

### Anti-PMD Inhibitors Bind to PfPLPs

**Figure 3:**
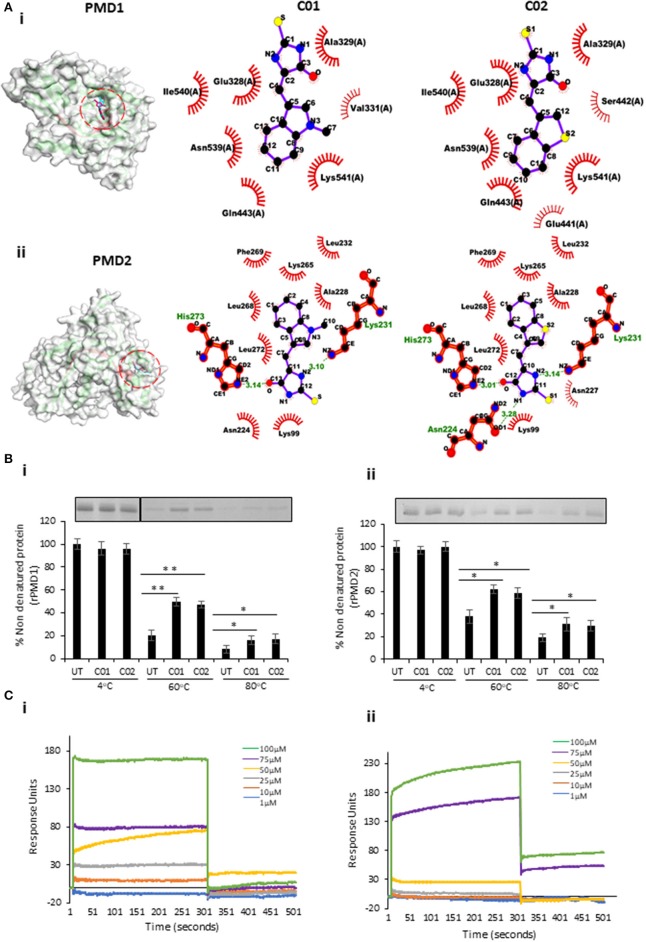
*In silico* and *in vitro* interaction of C01 and C02 with rPMDs. **(A)** The PyMOL rendered surface structures of docked complexes have shown strong binding of C01 and C02 to the signature motif of PMD1 (i) and PMD2 (ii). The ligplot+ rendered scheme demonstrates strong interactions as shown by the close proximity between the C01 and C02 and hydrophobic amino acids (depicted by bold, eyelash-like structures), and hydrogen bonds formed with polar residues. **(B)** Interaction of PMIs and PMDs by CETSA. The drug-target engagement between the compounds and recombinant proteins was analyzed by subjecting the samples to thermal denaturation at 60 and 80°C (i) (ii). The protein intensity at 4°C was taken as control. The band intensities graph was plotted considering the 4°C samples as 100% non-denatured protein. UT indicates PMI untreated sample. Error bar represents SD (*p < 0.05; **p < 0.01). **(C)** rPMD1 was immobilized onto a nickel charged NTA SPR chip. C01 (i) and C02 (ii) were injected over immobilized rPMD1. The PMIs show concentration-dependent binding to rPMD1 with a *K*_*d*_ value of 0.1183 ± 0.0370 and 0.0866 ± 0.0709 for C01 and C02, respectively.

To validate the binding of PMIs to PMDs *in silico* docking analysis was performed. The structure-refined models of PMD1-5 having an RMSD score of <2.0 were used for docking with PMIs. The molecular docking results revealed that C01 and C02 could efficiently bind to PMD1-5 as evident from strongly stable docking energy scores (Figure 3Ai,ii, Figures S6Ai–iii,B). The similar binding energy suggests that PMIs can inhibit PMDs with identical activity *in vitro*. *In silico* data further revealed that PMIs are binding in the same pocket of all PMDs. This pocket is not only structurally conserved but also has sequence identity as revealed by sequence alignment of PfPLP1-5 (Figure S6C).

To evaluate the binding of PMI to the rPMD, we modified and performed cellular thermal shift assay (CETSA). This technique involves the detection of a target protein by monitoring the thermostability of native protein in the presence of its selective inhibitor (Hashimoto et al., 2018). In principle, specific binding of the drug to its target protein increases the stability of protein at high temperatures (Jafari et al., 2014). In a similar line, we treated the purified rPMD1 and rPMD2 with C01 and C02 and heated them at 60 and 80°C while the sample at 4°C, served as a loading control. Analysis of band intensities demonstrated significant thermal protection of both rPMD1 and rPMD2 in the presence of compounds suggesting that C01 and C02 are interacting with rPMDs (Figure 3Bi,ii). Further, the interaction of PMIs to PMDs is strong enough to impart thermal protection to the recombinant protein.

Surface Plasmon Resonance (SPR) is a sensitive technique to validate the protein-protein as well as protein-drug interactions (Vuignier et al., 2010; Frostell et al., 2013). To further confirm the interaction of PMIs with rMAC1, we performed SPR with different compound concentrations (1–100 μM). In both cases, the compounds showed concentration-dependent binding to the coated rMAC1 (Figure 3Ci,ii). Together, SPR and CETSA confirm the binds of PMIs to rPMD.

### PMIs Inhibit PMD Mediated Pore Formation

**Figure 4:**
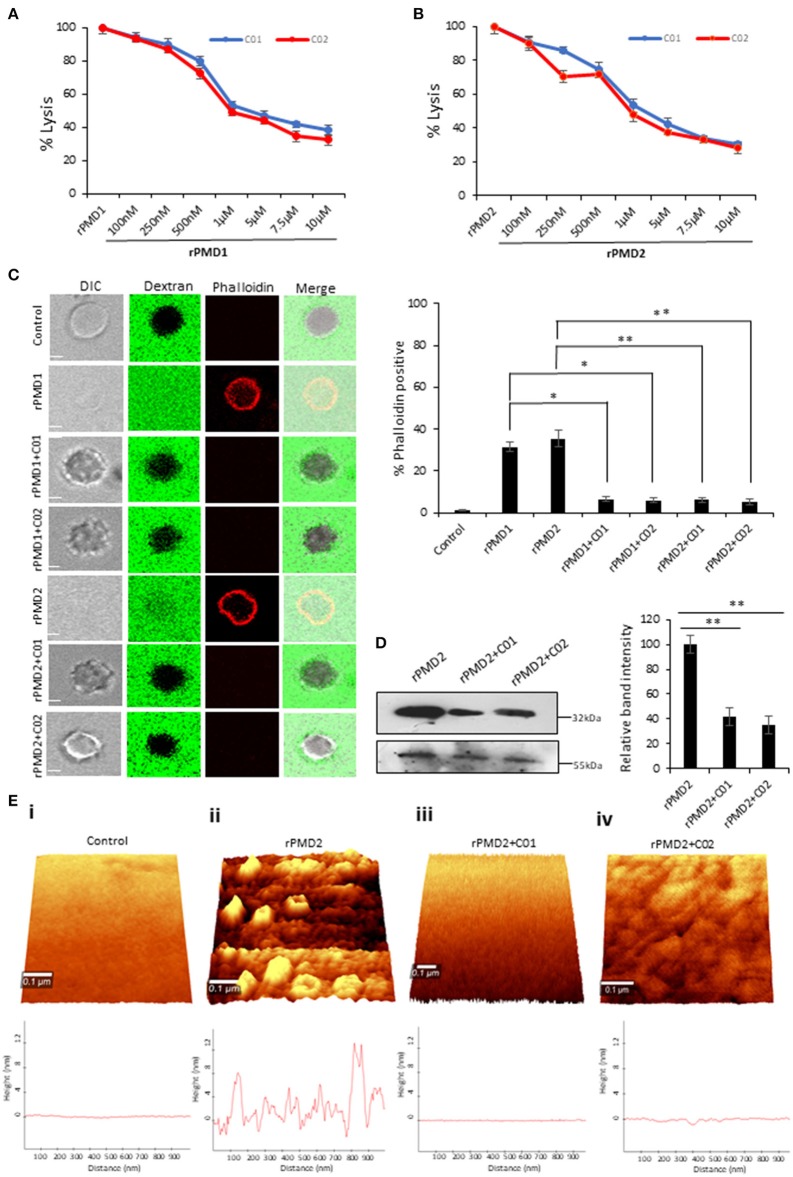
C01 and C02 rescue RBCs from rPMD mediated lysis. **(A,B)** Anti-PLP effect of C01 and C02. The inhibition in erythrocytes lysis was evaluated by treating different concentrations of compounds with EC_50_ (effective concentration of rPMD that causes 50% erythrocyte lysis) of rPMD1 (45 μg) **(A)** and rPMD2 (500 ng) **(B)**. The lysis caused by recombinant protein alone was considered as 100% and was plotted respectively for the compound treated erythrocytes (*n* = 3). The error bar represents SD. **(C)** Inhibition of permeabilization activity by PMIs. The erythrocytes were incubated with Rhodamine-phalloidin and 10 kDa FITC-dextran in the presence of recombinant proteins in the presence or absence of PMIs and visualized under the confocal microscope. PMI treatment inhibited phalloidin staining and dextran entry into the erythrocytes. The phalloidin positive erythrocytes were counted under the microscope for different treatments and their relative percentage was plotted. Error bar represents SD (*p < 0.05; **p < 0.01). **(D)** Inhibition of PMD binding to erythrocyte membrane in the presence of PMI. Western blot was performed to detect the effect of PMIs on the binding of rPMD2 to the erythrocyte membrane. Membrane Palmitoylated Protein 1 (MPP1) was taken as the initial erythrocytes loading control. The relative band intensity of bound rPMD2 was analyzed using Image J. Error bar represents SD (**p < 0.01). **(E)** Erythrocytes were treated with rPMD2 in the presence and absence of PMIs and pore formation was observed under the AFM (*n* = 2). (ii) The PMD treated erythrocytes demonstrate the formation of pores on the erythrocyte surface. The line profile further depicts the roughness of erythrocytes. The PMD treated erythrocytes (iii, iv) in the presence of PMIs does not demonstrate the formation of pores and the surface roughness was also reduced to normal erythrocytes (i).

We analyzed the inhibitory activity of PMIs by performing rPMD mediated erythrocyte lysis assay in the presence of C01 and C02. The results demonstrated that C01 and C02 could inhibit the activity of both rPMD1 and rPMD2 in the sub-micromolar range (Figures 4A,B). Together, these data demonstrate the designing of anti-PMD inhibitors that inhibit the pore-forming ability of PMDs.

To investigate the complete disruption of pore formation in PMI treated erythrocytes, the influx of small molecules such as rhodamine-Phalloidin (~1 kDa) and FITC-dextran (10 kDa) was investigated. PMD treated erythrocytes could not uptake dextran or Phalloidin in presence of PMI as compared to PMI treated erythrocytes (Figure 4C). This confirms that there is a complete abrogation of pore formation in the presence of PMIs.

Since the pore formation is chiefly dependent on the binding of monomers, we tested the binding of monomer to erythrocyte membrane in the presence of PMIs. rPMDs could not bind to the erythrocyte membrane in the presence of PMIs suggesting that inhibition in pore formation is due to the restriction of monomer binding to erythrocyte membrane (Figure 4D). Next, to closely monitor that PMIs are completely abrogating formation of pores and there is little to no change in membrane roughness due to rPMD binding, AFM was performed to evaluate surface topology of treated erythrocytes. We could clearly detect the formation of pores on the erythrocyte membrane treated with rPMD2 (Figure 4Eii). Also, the surface of rPMD treated erythrocytes was very rough and uneven. In comparison, treatment with rPMD2 in presence of PMIs, displayed no evident structures on the surface of erythrocytes, indicating the impairment in oligomerization and subsequent pore formation (Figure 4Eiii,iv). Further, the smooth surface of PMI treated erythrocytes, similar to untreated erythrocytes, suggests that PMIs could revert all the phenotypes induced by rPMDs (Figure 4Ei–iv). Taken together, this data indicates that C01 and C02 inhibit pore formation by a decrease in binding of the rPMD to the erythrocyte membrane.

### PMIs Demonstrate Anti-parasitic Activity at Multiple Stages of Parasite Life Cycles

**Figure 5:**
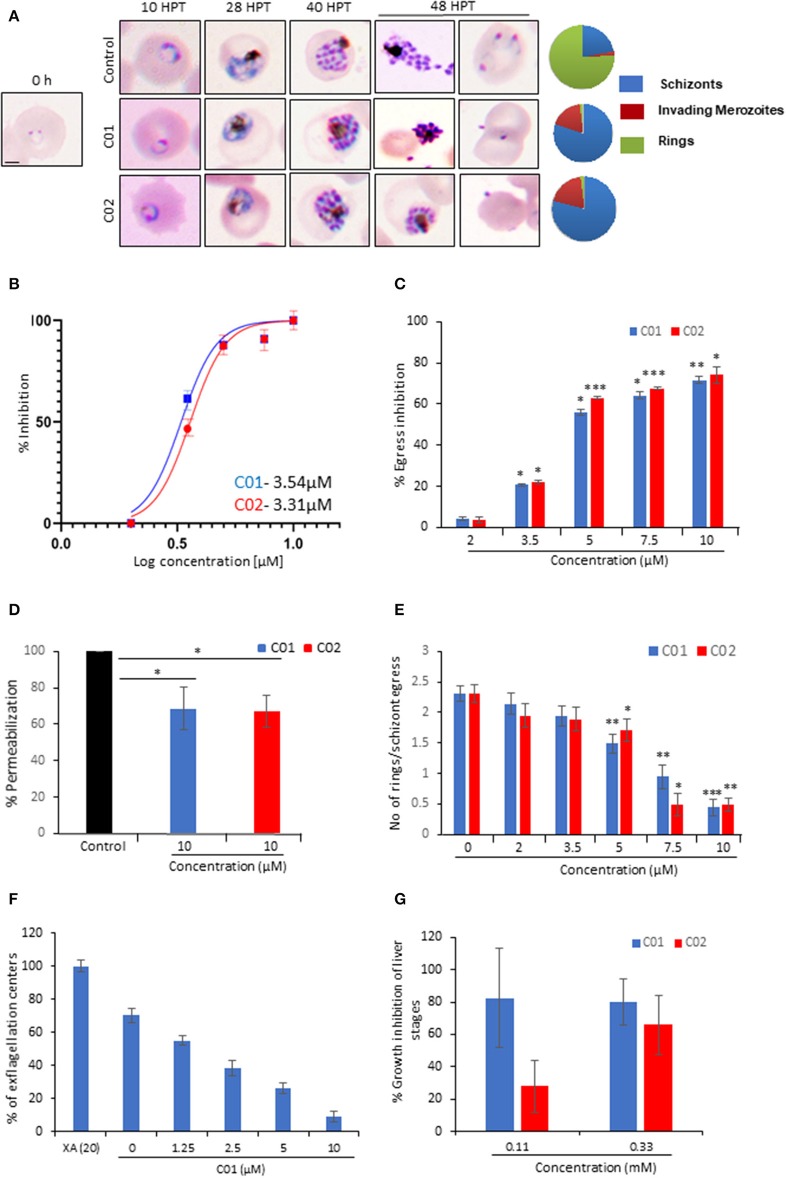
C01 and C02 show multistage inhibition. **(A)** Images of Giemsa stained smears of PMI treated (10 μM) and untreated parasites. Early rings were treated with PMIs and smears were prepared 10, 28, 40, and 48 h post-treatment (HPT). Pie charts show relative proportions of schizonts, invading merozoites and rings. **(B)** C01 and C02 inhibit the *in vitro* growth of *P. falciparum*. The effect of PMIs on growth inhibition of *P. falciparum* was evaluated after one cycle of parasite growth (*n* = 3). Scale bar represents 2 μm. **(C)** Late-stage schizonts were treated with different concentrations of compounds. The relative inhibition in egress was calculated by counting the number of remaining schizonts after 7 h of treatment as compared to control (*n* = 3). Error bar represents SD (*p < 0.05; **p < 0.01; ***p < 0.005). **(D)** Permeabilization of PMI-treated schizonts was measured by flow cytometry using Phalloidin. Error bar represents SD (*p < 0.05). **(E)** Late-stage schizonts were treated with different concentrations of PMIs. The ability to form rings per schizont egress was calculated by counting the number of schizonts and rings after 7 h using Giemsa stained smears (*n* = 3). Error bar represents SD (*p < 0.05; **p < 0.01; ***p < 0.005). **(F)** Treatment of male gametocytes with PMIs inhibits exflagellation. The number of exflagellation centers was scored 15 min post-activation in 30 optical fields at 40X magnification by light microscopy. Exflagellation efficiency of PMI treated vs. untreated gametocytes is shown (*n* = 2). **(G)** HepG2 cells were infected with *P. berghei* sporozoites and treated with PMIs. Parasite growth was assessed after 51 h via real-time PCR using *P. berghei* 18S rRNA specific primers. Parasite growth inhibition was calculated by dividing the 18S rRNA copy number of the experimental group by that of the untreated control group. The fraction obtained was then converted into % inhibition (with respect to untreated as 100%) (*n* = 2).

To monitor the effect of PMIs on parasite growth, Giemsa stained smears were prepared for the treated as well as untreated parasites at different time points. PMI treatment (10 μM) did not affect the parasite growth from ring to schizont stages. However, the treated parasites could not egress, and the merozoites that came out could be seen attached to erythrocytes but not forming rings (Figure 5A). Having confirmed the inhibitory activity of PMIs we further evaluated their anti-malarial activity. The ring infected parasites (~5–6 hpi) were treated with different concentrations of drugs for 72 h. The parasites treated with DMSO served as a control and the parasite growth was measured by fluorimetry using SYBR green I-based assay. Both C01 and C02 demonstrated the ability to inhibit parasite growth with an IC_50_ of 3.54 μM for C01 and 3.31 μM for C02 (Figure 5B). This is similar to the reported role of PfPLPs in egress and invasion of merozoites suggesting the specific action of PMIs toward PMDs.

To confirm the egress defect, late schizonts (~44–46 hpi) were treated with different concentrations of C01 and C02 for 6 h and a decrease in the number of schizonts was scored (Figure 5C). The intracellular Ca^2+^ chelator BAPTA-AM was used as a positive control. We observed a dose-dependent defect in egress. PfPLPs have a role in host erythrocyte permeabilization that facilitates the egress of merozoites. We observed that PMI treated schizonts demonstrated restriction in permeabilization of host erythrocyte as compared to untreated schizonts (Figures 5C,D), implying specific action of PMIs toward PfPLPs in egress.

Since the Giemsa scoring also suggested a defect in the invasion, we scored for ring formation after 10 h post-treatment. To specifically assess the effects of C01 and C02 on invasion and avoid the effects of their influence on egress, we calculated the number of rings formed per schizont egress. A drastic decrease in the number of rings formed in treated parasites as compared to the untreated control was noted (Figure 5E). To check the possibility of any non-specific effect of PMIs on erythrocytes, PMIs treated erythrocytes were incubated with purified schizonts and the parasite growth was accessed after 8 h. The data indicates no significant difference in parasite invasion to the untreated and the PMI erythrocytes, indicating that PMIs do not have any non-specific effect on erythrocytes (Figure S7A).

Further, to rule out the possibility that PMI treatment has any effect on ring and trophozoite stages, we devised a ring toxicity assay and trophozoite toxicity assay. In these assays, rings or trophozoites were treated for 6 h, washed and accessed for their growth during the next cycle. If the compounds have any toxicity on these stages, it will be reflected in this assay. Our data suggest that these compounds do not exhibit any toxicity toward either ring and trophozoite stages (Figures S7B,C). Taken together these findings imply that both compounds act only on the stages which involve the PPLP activity and can be used as a generic inhibitor of PPLPs.

Since PPLPs play a role during multiple stages of the parasite cycle, we studied their cross-stage inhibitory potential. PPLP2 plays a role in gametocyte egress (Deligianni et al., 2013; Wirth et al., 2015) and hence the effect of PMI was monitored during this stage. The egress efficiency of male gametocytes, as revealed by counting exflagellation centers, was markedly reduced following PMI treatment (Figure 5F). To test the inhibitory effect of PMI on sporozoite infection, we infected HepG2 cells with *P. berghei* sporozoites treated with different concentrations of PMI for 72 h. The sporozoite infection was scored using real-time PCR analysis. As shown (Figure 5G), both compounds displayed an inhibitory effect toward *P. berghei* sporozoites. Both the compounds showed no defect in the viability of HepG2 cells suggesting the specific effect toward sporozoite but not host (Figure S7D).

Together, this suggests the cross-stage anti-malarial activity of PMIs which can be further exploited to deliver both symptomatic and transmission-blocking cure for the disease.

## Discussion

Despite abundant evidence that PPLPs are crucial for the life cycle progression of malaria parasites, the development of chemotherapeutic interventions against them has not been explored. In the blood stage of *P. falciparum*, PfPLP1 and PfPLP2 create pores on the erythrocyte membrane that help in the exit of merozoites (Garg et al., 2013). For the development of pan-active anti-PLP therapeutics, we first identified central, pan-active motif of PfPLPs, PMD domain, that is highly conserved across all *Plasmodium* spp (Figure S3) and oligomerize on host membranes to create pores (Pipkin and Lieberman, 2007; Baran et al., 2009). To visualize pore formation at physiological relevance, AFM was performed with a modified protocol to overcome the limitations associated with the non-adherent, rough cell surface of erythrocyte. The modified protocol could successfully demonstrate that rPMD2 drilled pores of 10.55 nm ± 3.22 nm height and 24.33 ± 7.42 nm width on the erythrocytes (Figure 1H). The characteristics of the pore formed by PfPLP are similar to earlier reported eukaryotic PFPs which form smaller pores as compared to prokaryotic PFPs which form larger pores (Metkar et al., 2015). The three-dimensional topology not only confirmed the presence of well-defined pores but also revealed that the rPMD2 treated erythrocyte surface is much rougher and more uneven as compared to the untreated erythrocytes (Figure 1Hi,ii). The roughness of erythrocytes reflects its innate response toward any non-physiological, toxic agent which in this case is rPMD2. This information about the cellular response is usually missed in other studies where they studied pore formation on model membranes.

Given the multi-step process of pore formation by PLP, there exist multiple stages at which a PLP can be targeted for inhibition. The designed PMIs strongly bind to the PMD domain and inhibits its pore forming activity by restricting the initial attachment of the monomer to the erythrocyte surface (Figure 4D). The decreased binding of monomer inhibits the oligomerization of PMD to form pores confirming that PMIs completely abrogated the activity of PfPLPs. Ring stage parasites treated with PMIs progresse to schizonts without any growth defect, but the mature parasites could not egress and re-invade to new erythrocyte (Figures 5A,C,D). This finding is in line with the role of PfPLPs in schizont egress and merozoite invasion and suggests that PMIs are not showing any off-target effect *in vivo*. Furthermore, PMIs inhibited gametogenesis and sporozoite infection to HepG2 cells validating their potential in blocking the transmission of malaria parasites (Figures 5F,G). The invasion inhibitory activity of PMIs against sporozoite of *P. berghei* is suggestive of their cross-species activity due to evolutionary conservation and high similarity of PMDs. These scaffolds could be improved for their efficacy and development of better antimalarials.

In this study, we further propose that the secretion of PfPLPs in the blood leads to the formation of pores on bystander erythrocytes leading to their premature senescence and identifies it as one of the probable cause for anemia in severe malaria. This phenomenon was similar to earlier reports where it has been shown that binding of some parasite proteins to erythrocytes leads to anemia (Haldar and Mohandas, 2009; Gómez et al., 2011). But the mechanistic role underlying the induction of anemia remains to be fully elucidated. However, our data defines that the smaller pores created by PfPLPs induce calcium influx (Figure 2A-a) in erythrocytes leading to phosphatidylserine exposure (Figure 2B), as a signal for recognition by macrophages. This is in line with earlier studies that have demonstrated that an increase in intracellular calcium induces premature senescence of erythrocytes (Rettig et al., 1999; Arashiki et al., 2013).

The enhanced oxidative stress in erythrocyte senescence is represented by the formation of the oxidized form of hemoglobin, i.e., methemoglobin (Figures 2D,E) (Umbreit, 2007; Pamplona et al., 2009). The increased levels of methemoglobin *in vivo* are toxic to the plasma lipoproteins, endothelial cells, and other vascular organs. Therefore, the erythrocytes with increased methemoglobin concentrations are cleared from the body through spleen which is a cause of anemia in methemoglobinemia (Wagener et al., 2001; Umbreit, 2007). The presence of Fe^3+^ heme form in methemoglobin causes changes in pyrrole ring breathing and asymmetrical pyrrole half-ring stretching vibrations (Dybas et al., 2018). Using Raman spectroscopy, we could detect the presence of these metabolic signatures in erythrocytes after 48 h of PfPLP treatment representing the formation of methemoglobin (Figure 2E). The earlier clinical reports advocate the identification of elevated levels of methemoglobin in severe malaria and directly correlates it with the death of patients (Behera et al., 2016). Our study supports the role of PfPLP in inducing premature senescence of erythrocytes leading to anemia in severe malaria.

Taken together, we identified novel pharmacological inhibitors against PPLPs that can preserve the cell membrane integrity and viability of all host cells and thus aid in facilitating parasite elimination by preventing further infection. Also, the proposed therapy can also be used as anti-virulence therapy along with routine medication for improving treatment outcomes. Since the anti-virulence strategies do not focus on directly killing the pathogen, the parasite is not under the selective pressure of resistance development. Therefore, our study lends weight for the development of novel pharmacological approaches against malaria to not only cure the disease but also ameliorate disease pathogenesis in clinical conditions.

## Data Availability Statement

The raw data supporting the conclusions of this article will be made available by the authors, without undue reservation, to any qualified researcher.

## Author Contributions

AS and SG performed the recombinant protein and inhibitors based *in vitro* and *in vivo* assays. SSe and CB synthesized and characterized the inhibitors. RH helped to design and perform *in vitro P. falciparum* assays. SP and RA performed the *in silico* experiments and data analysis. SG, AS, APS, and IK performed the liver stage inhibition assay. SSi performed the gametocyte inhibition assay. SSi and SG conceived and designed the experiments. AS, SG, APS, and SSi analyzed the data and wrote the manuscript. All the authors commented on the manuscript.

### Conflict of Interest

The authors declare that the research was conducted in the absence of any commercial or financial relationships that could be construed as a potential conflict of interest.

## References

[B1] Alaganan A. Singh P. Chitnis C. E. (2017). Molecular mechanisms that mediate invasion and egress of malaria parasites from red blood cells. Curr. Opin. Hematol. 3, 208–214. 10.1097/MOH.0000000000000334 28306665

[B2] Arashiki N. Kimata N. Manno S. Mohandas N. Takakuwa Y. (2013). Membrane peroxidation and methemoglobin formation are both necessary for band 3 clustering: mechanistic insights into human erythrocyte senescence. Biochemistry 52, 5760–5769. 10.1021/bi400405p 23889086 PMC3914982

[B3] Baran K. Dunstone M. Chia J. Ciccone A. Browne K. A. Clarke C. J. P.. (2009). The molecular basis for perforin oligomerization and transmembrane pore assembly. Immunity 30, 684–695. 10.1016/j.immuni.2009.03.016 19446473

[B4] Barkur S. Mathur D. Chidangil S. (2018). A laser raman tweezers study of eryptosis. J. Raman Spectrosc. 49, 1155–1164. 10.1002/jrs.5361

[B5] Behera G. C. Behera S. K. Jena R. K. Bharati V. S. (2016). Study of methaemoglobin in malaria patients. Indian J. Hematol. Blood Transfus. 32, 100–103. 10.1007/s12288-015-0522-5 26855515 PMC4733672

[B6] Brown G. C. Neher J. J. (2012). Eaten alive! cell death by primary phagocytosis: Phagoptosis. Trends Biochem. Sci. 37, 325–32. 10.1016/j.tibs.2012.05.002 22682109

[B7] Callahan M. K. Halleck M. S. Krahling S. Henderson A. J. Williamson P. Schlegel R. A. (2003). Phosphatidylserine expression and phagocytosis of apoptotic thymocytes during differentiation of monocytic cells. J. Leukoc. Biol. 74, 846–856. 10.1189/jlb.0902433 12960250

[B8] Deligianni E. Morgan R. N. Bertuccini L. Wirth C. C. Silmon de Monerri N. C. Spanos L.. (2013). A perforin-like protein mediates disruption of the erythrocyte membrane during egress of *Plasmodium berghei* male gametocytes. Cell. Microbiol. 15, 1438–1455. 10.1111/cmi.12131 23461714

[B9] Dybas J. Grosicki M. Baranska M. Marzec K. M. (2018). Raman imaging of heme metabolism: *in situ* in macrophages and Kupffer cells. Analyst. 143, 3489–3498. 10.1039/C8AN00282G 29951676

[B10] Ecker A. Pinto S. B. Baker K. W. Kafatos F. C. Sinden R. E. (2007). Plasmodium berghei: plasmodium perforin-like protein 5 is required for mosquito midgut invasion in *Anopheles stephensi*. Exp. Parasitol. 116, 504–508. 10.1016/j.exppara.2007.01.015 17367780 PMC1916484

[B11] Frostell Å. Vinterbäck L. Sjöbom H. (2013). Protein-ligand interactions using SPR systems. Methods Mol. Biol. 1008, 139–165. 10.1007/978-1-62703-398-5_6 23729252

[B12] Garg S. Agarwal S. Kumar S. Shams Yazdani S. Chitnis C. E. Singh S. (2013). Calcium-dependent permeabilization of erythrocytes by a perforin-like protein during egress of malaria parasites. Nat. Commun. 4:1736. 10.1038/ncomms2725 23591903

[B13] Garg S. Sharma V. Ramu D. Singh S. (2015). *In silico* analysis of calcium binding pocket of perforin like protein 1: insights into the regulation of pore formation. Syst. Synth. Biol. 9(Suppl.1):17–21. 10.1007/s11693-015-9166-x 26702304 PMC4688409

[B14] Garg S. Shivappagowdar A. Hada R. S. Ayana R. Bathula C. Sen S. (2019). Supression of perforin-like protein pores inhibit *plasmodium* multistage-growth, transmission and erythrocyte senescence. bioRxiv 756197 10.1101/756197

[B15] Gaur D. Singh S. Singh S. Jiang L. Diouf A. Miller L. H. (2007). Recombinant *Plasmodium falciparum* reticulocyte homology protein 4 binds to erythrocytes and blocks invasion. Proc. Natl. Acad. Sci. U. S. A. 104, 17789–17794. 10.1073/pnas.0708772104 17971435 PMC2077073

[B16] Gilbert R. J. C. Stansfeld P. J. Harlos K. Ni T. Rezelj S. Williams S. I.. (2018). Structures of monomeric and oligomeric forms of the *Toxoplasma gondii* perforin-like protein 1. Sci Adv. 4:eaaq0762. 10.1126/sciadv.aaq0762 29750191 PMC5943054

[B17] Gómez N. D. Safeukui I. Adelani A. A. Tewari R. Reddy J. K. Rao S.. (2011). Deletion of a malaria invasion gene reduces death and anemia, in model hosts. PLoS ONE 6:e25477. 10.1371/journal.pone.0025477 21980474 PMC3182240

[B18] Hadders M. A. Beringer D. X. Gros P. (2007). Structure of C8alpha-MACPF reveals mechanism of membrane attack in complement immune defense. Science 317, 1552–1554. 10.1126/science.1147103 17872444

[B19] Haldar K. Mohandas N. (2009). Malaria, erythrocytic infection, and anemia. Hematol. Am. Soc. Hematol. Educ. Program 2009, 87–93. 10.1182/asheducation-2009.1.87 PMC2933134 20008186

[B20] Hashimoto M. Girardi E. Eichner R. Superti-Furga G. (2018). Detection of chemical engagement of solute carrier proteins by a cellular thermal shift assay. ACS Chem. Biol. 13, 1480–1486. 10.1021/acschembio.8b00270 29851333 PMC6067815

[B21] Ishino T. Chinzei Y. Yuda M. (2005). A *Plasmodium sporozoite* protein with a membrane attack complex domain is required for breaching the liver sinusoidal cell layer prior to hepatocyte infection. Cell. Microbiol. 7, 199–208. 10.1111/j.1462-5822.2004.00447.x 15659064

[B22] Jafari R. Almqvist H. Axelsson H. Ignatushchenko M. Lundbäck T. Nordlund P.. (2014). The cellular thermal shift assay for evaluating drug target interactions in cells. Nat. Protoc. 9, 2100–2122. 10.1038/nprot.2014.138 25101824

[B23] Kadota K. Matsuyama T. Ishino T. Yuda M. Chinzei Y. (2004). From the cover: essential role of membrane-attack protein in malarial transmission to mosquito host. Proc. Natl. Acad. Sci. U. S. A. 101, 16310–16315. 10.1073/pnas.0406187101 15520375 PMC524694

[B24] Kafsack B. F. C. Pena J. D. O. Coppens I. Ravindran S. Boothroyd J. C. Carruthers V. B. (2009). Rapid membrane disruption by a perforin-like protein facilitates parasite exit from host cells. Science 323, 530–533. 10.1126/science.1165740 19095897 PMC2662845

[B25] Kataoka T. Taniguchi M. Yamada A. Suzuki H. Hamada S. Magae J.. (1996a). Identification of low molecular weight probes on perforin- and fas-based killing mediated by cytotoxic T lymphocytes. Biosci. Biotechnol. Biochem. 60, 1726–1728. 10.1271/bbb.60.1726 8987676

[B30] Kataoka T. Shinohara N. Takayama H. Takaku K. Kondo S. Yonehara S.. (1996b). Concanamycin A, a powerful tool for characterization and estimation of contribution of perforin-and Fas-based lytic pathways in cellmediated cytotoxicity. J Immunol. 156, 3678–3686. 8621902

[B26] Lasonder E. Ishihama Y. Andersen J. S. Vermunt A. M. W. Pain A. Sauerwein R. W.. (2002). Analysis of the *Plasmodium falciparum* proteome by high-accuracy mass spedrometry. Nature 419, 537–542. 10.1038/nature01111 12368870

[B27] Lena G. Trapani J. A. Sutton V. R. Ciccone A. Browne K. A. Smyth M. J.. (2008). Dihydrofuro[3,4-c]pyridinones as inhibitors of the cytolytic effects of the pore-forming glycoprotein perforin. J. Med. Chem. 51, 7614–7624. 10.1021/jm801063n 19007200

[B28] Metkar S. S. Marchioretto M. Antonini V. Lunelli L. Wang B. Gilbert R. J.. (2015). Perforin oligomers form arcs in cellular membranes: a locus for intracellular delivery of granzymes. Cell Death Differ. 22, 74–85. 10.1038/cdd.2014.110 25146929 PMC4262768

[B29] Miller C. K. Huttunen K. M. Denny W. A. Jaiswal J. K. Ciccone A. Browne K. A.. (2016). Diarylthiophenes as inhibitors of the pore-forming protein perforin. Bioorg. Med. Chem. Lett. 26, 355–360. 10.1016/j.bmcl.2015.12.003 26711151 PMC4706532

[B31] Pamplona A. Hanscheid T. Epiphanio S. Mota M. M. Vigário A. M. (2009). Cerebral malaria and the hemolysis/methemoglobin/heme hypothesis: shedding new light on an old disease. Int. J. Biochem. Cell Biol. 41, 711–716. 10.1016/j.biocel.2008.09.020 18930163

[B32] Pipkin M. E. Lieberman J. (2007). Delivering the kiss of death: progress on understanding how perforin works. Curr. Opin. Immunol. 19, 301–308. 10.1016/j.coi.2007.04.011 17433871 PMC11484871

[B33] Ramu D. Garg S. Ayana R. Keerthana A. K. Sharma V. Saini C. P.. (2017). Novel β-carboline-quinazolinone hybrids disrupt *Leishmania donovani* redox homeostasis and show promising antileishmanial activity. Biochem. Pharmacol. 129, 26–42. 10.1016/j.bcp.2016.12.012 28017772

[B34] Rettig M. P. Low P. S. Gimm J. A. Mohandas N. Wang J. Christian J. A. (1999). Evaluation of biochemical changes during *in vivo* erythrocyte senescence in the dog. Blood 93, 376–384. 10.1182/blood.V93.1.376.401k41_376_384 9864184

[B35] Rosado C. J. Buckle A. M. Law R. H. P. Butcher R. E. Kan W. T. Bird C. H.. (2007). A common fold mediates vertebrate defense and bacterial attack. Science 317, 1548–1551. 10.1126/science.1144706 17717151

[B36] Spicer J. A. Lena G. Lyons D. M. Huttunen K. M. Miller C. K. O'Connor P. D.. (2013). Exploration of a series of 5-arylidene-2-thioxoimidazolidin-4-ones as inhibitors of the cytolytic protein perforin. J. Med. Chem. 56, 9542–9555. 10.1021/jm401604x 24195776 PMC3865801

[B37] Tavares J. Amino R. Ménard R. (2014). The role of MACPF proteins in the biology of malaria and other apicomplexan parasites. Subcell. Biochem. 80, 241–253. 10.1007/978-94-017-8881-6_12 24798015

[B38] Trager W. Jensen J. B. (1976). Human malaria parasites in continuous culture. Science 193:673–675. 10.1126/science.781840 781840

[B39] Umbreit J. (2007). Methemoglobin—it's not just blue: a concise review. Am. J. Hematol. 82, 134–144. 10.1002/ajh.20738 16986127

[B40] Voskoboinik I. Thia M. C. Fletcher J. Ciccone A. Browne K. Smyth M. J. (2005). Calcium-dependent plasma membrane binding and cell lygis by perforin are mediated through its C2 domain: a critical role for aspartate residues 429, 435, 483, and 485 but not 491. J. Biol. Chem. 280, 8426–8434. 10.1074/jbc.M413303200 15576364

[B41] Vuignier K. Schappler J. Veuthey J. L. Carrupt P. A. Martel S. (2010). Drug-protein binding: a critical review of analytical tools. Anal. Bioanal. Chem. 398, 53–66. 10.1007/s00216-010-3737-1 20454782

[B42] Wagener F. A. Eggert A. Boerman O. C. Oyen W. J. Verhofstad A. Abraham N. G.. (2001). Heme is a potent inducer of inflammation in mice and is counteracted by heme oxygenase. Blood 98, 1802–1811. 10.1182/blood.V98.6.1802 11535514

[B43] WHO (2018). World Malaria Report 2018. Geneva: World Health Organization.

[B44] Wirth C. C. Bennink S. Scheuermayer M. Fischer R. Pradel G. (2015). Perforin-like protein PPLP4 is crucial for mosquito midgut infection by *Plasmodium falciparum*. Mol. Biochem. Parasitol. 201, 90–99. 10.1016/j.molbiopara.2015.06.005 26166358

[B45] Wirth C. C. Glushakova S. Scheuermayer M. Repnik U. Garg S. Schaack D.. (2014). Perforin-like protein PPLP2 permeabilizes the red blood cell membrane during egress of *Plasmodium falciparum* gametocytes. Cell. Microbiol. 16, 709–733. 10.1111/cmi.12288 24602217 PMC4312913

[B46] Yang A. S. P. O'Neill M. T. Jennison C. Lopaticki S. Allison C. C. Armistead J. S.. (2017). Cell traversal activity is important for *Plasmodium falciparum* liver infection in humanized mice. Cell Rep. 18, 3105–3116. 10.1016/j.celrep.2017.03.017 28355563

