# Supplementary material for: Plasmodium Perforin-Like Protein Pores on the Host Cell Membrane Contribute in Its Multistage Growth and Erythrocyte Senescence
Source: Front Cell Infect Microbiol. 2020 Mar 24;10:121. doi: 10.3389/fcimb.2020.00121 (PMC7105882; doi:10.3389/fcimb.2020.00121)
Supplement: Supplementary file 1 [file Data_Sheet_1.PDF]

Supplementary Material

A

ATCATCAGCAAGCATACCAGCGTTTTTCCGGGTCTGTACTTCATCGGCATCGGCTATAACCTGCTGTTTGGT
AACCCGCTGGGTGAAGCGGACAGCCTGATTGATCCGGGTTACCGTGCGCAGATTTATCTGATGGAGTGGGCG
CTGAGCAAGGAAGGTATCGCGAACGACCTGAGCACCCTGCAACCGGTGAACGGCTGGATTCGTAAAGAAAAC
GCGTGCAGCCGTGTTGAGAGCATCACCGAATGCAGCAGCATTAGCGATTACACCAAGAGCCTGAGCGCGGAG
GCGAAAGTGAGCGGTAGCTACTGGGGTATTGCGAGCTTCAGCGCGAGCACCGGCTATAGCAGCTTTCTGCAC
GAAGTTACCAAGCGTAGCAAGAAAACCTTCCTGGTGAAGAGCAACTGCGTTAAATACACCATCGGTCTGCCG
CCGTATATTCCGTGGGACAAGACCACCGCGTACAAAAACGCGGTGAACGAACTGCCGGCGGTTTTTACCGGC
CTGGACAAGGAGAGCGAATGCCCGAGCGATGTGTATGAGGAAAACAAGACCAAAAGCAACTGCGAGAACGTT
AGCCTGTGGATGAAATTCTTTGATATCTACGGTACCCACATCATTTATGAAAGCCAGCTGGGTGGCAAGATC
ACCAAAATCATTAACGTGAGCACCAGCAGCATTGAGCAAATGAAGAAAAACGGCGTGAGCGTTAAGGCGAAA
ATTCAGGCGCAATTCGGTTTTGGCAGCGCGGGTGGCAGCACCGACGTTAACAGCAGCAACAGCAGCGCGAAC
GACGAGCAGAGCTATGATATGAACGAACAACTGATCGTGATTGGTGGCAACCCGATCAAGGATGTTACCAAA
GAGGAAAACCTGTTCGAGTGGAGCAAGACCGTGACCAACCACCCGATGCCGATCAACATTAAACTGACCCCG
ATTAGCGACAGCTTTGATAGCGACGATCTGAAAGAAAGCTACGACAAAGCGATTATTTACTATAGCCGCCTG
TACGGTCTGAGCCCGCACGAC

B

ATGAAACCGTGGGTGATCCCGGAACACTCGTGCTCGCAATCTAAAAATGTGGAAGAAATTCGTAATCTGGAA
CAATACAAACTGGAACTGCTGTCGGATGTGAAAGTTAGCACGCCGAGCTCTTTTCCGTATTCATTCTCGGCG
AGCGCCGAATTTAAAAACGCGCTGAAAAAACTGAAAGTCCAGAACAACGTGATCTTCCTGATGAAAATCTAC
TGCCTGCGTTATTACACCGGCATCCCGATTACCACGACCTCCTACAAATTCTCAGAAAACTTCAAAAACGCC
CTGAGCAAACTGCCGAAATATTTTGATGGTCTGCGCGAAGACTCTAAATGCAGTTACGAATACTACATCAAC
AAACTGAACAGTCCGGAATGTGAAGAAAACGTGAATAAATGGATGCTGTTTTTCAAACTGCATGGCACGCAC
GTTGCATATGAAATTTACCTGGGCGGTAAAATCATCATCAAAATCAACATCAACAAAGAAGAATATAACAAA
ATGAAAGAAAACAACATCAACGTCAAAACCTTTTTCAACATCTACTTCCATAAAATGGGCCTGAGTTCCGCA
TTCCAGAAAGAAGCTCAGAAAATCCTGAACAAATTCCGTATCTCCAAACATATCGCCATTCTGGGCGGTAAC
CCGGGTCTGAACGTTAACAACACCAGCTTTTTCGAAAAATGGGTCCACTCTATCAACACGAATAGTATGCCG
ATTCGCACCAAACTGCTGCCGTTTAGCTTTTTCATGGACGATAATGATATGATTCAAGCCTACAAAGATGCT
CTGATTTTCTATGGCCTGACCTACGGC

Supplementary Figure S1. Codon optimized sequence. The codon optimized sequences for rPMD1 (**A**) and rPMD2 (**B**) are represented.

A

```
PfPLP1          NACSRVESITECSSISDYTKSLSAEAKVSGSYWGIASFSASTGYSSFLHEVTKRSKKTFL
PfPLP3          -SCNRSEKESEISTMSEYTKELSVDASIGASYGLFGSFSASTGYKSVSNTISKNKFRMFM
PfPLP2          HSCSQSKNVEEIRNLEQYKLELLSDVKVSTPSSFPYSFSASAEFKNALKKLKVQNNVIFL
PfPLP5          IKCIDTIKENVIDNLCDINKEYERSYSVSSINDDIHPFNDSNYYKMLVKRIN-RGDSIII
PfPLP4          -ICKKEEYVDVIEDINDIGYLGMQKINIDDLDNRIKPFSASMPYKSYFADLEIKKKKYAL
                  *           : :       . .:.       .*. *  :.     :  .     :

PfPLP1          VKSNCVKYTIGLPPYIP-WDKTTAYKNAVNELPAVFTGLDKE-SECPSDVYEENKT---K
PfPLP3          LKSYCFKYVASLSQYSQ-WKLTDQFVRAISLLPSHFNSLEKDGTYCSDEEFRDNRK---S
PfPLP2          MKIYCLRYYTGIPITTTSYKFSENFKNALSKLPKYFDGLRED-SKCSYEYYINKLN---S
PfPLP5          EKKLCSKYFSFINDINK-NDLDTFFLTTLNELGDNYQNIKDDTYKCSLQYYKMNNMNKYS
PfPLP4          AQNMCVLNYATYDLKESGNNINKDFVLDIEKLPILTKNQMKL---CTKVLYMNNNL----
                 :  *              .    :   :. *     .  .    *.   :  :

PfPLP1          SNCEN-VSLWMKFFDIYGTHIIYESQLGGKITKIINVSTSSIEQMKKNGVSVKAKIQAQF
PfPLP3          EKCGKSVTAWMYFFKNFGTHVSTLLHLGGKITQQVKISKNDYKAMTESGLSISASVSAGF
PfPLP2          PECEENVNKWMLFFKLHGTHVAYEIYLGGKIIIKININKEEYNKMKENNINVKTFFNIYF
PfPLP5          ENCLKTITPWISFFNMYGTHVISGVYYGGKIIHNLYFENNNLKKKEYKIRMYKSRLNPFS
PfPLP4          -HCSEGIKSWMKFFEKYGTHVVLSAHFGGMSFNTMEITKRKIEEIKIYKYKYSLWNNPYL
                 .* : :. *: **. .***:      **     : . . . :         .   .

PfPLP1          GFGSAGG
PfPLP3          GLFKVKG
PfPLP2          HKMGLSS
PfPLP5          TINSN--
PfPLP4          NIFKS--
```

B

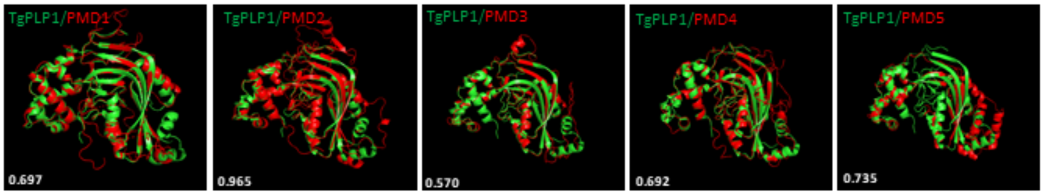

Supplementary Figure S2. Sequence and structural alignment of the MAC domains of PfPLP1-5. (**A**) The ClustalW alignment of MACPF domains of PfPLP1-5. Conserved residues are highlighted in yellow. (**B**) The structural superimposition of PMDs with the MACPF domain of TgPLP1 is shown. The RMSD values are indicated in white.

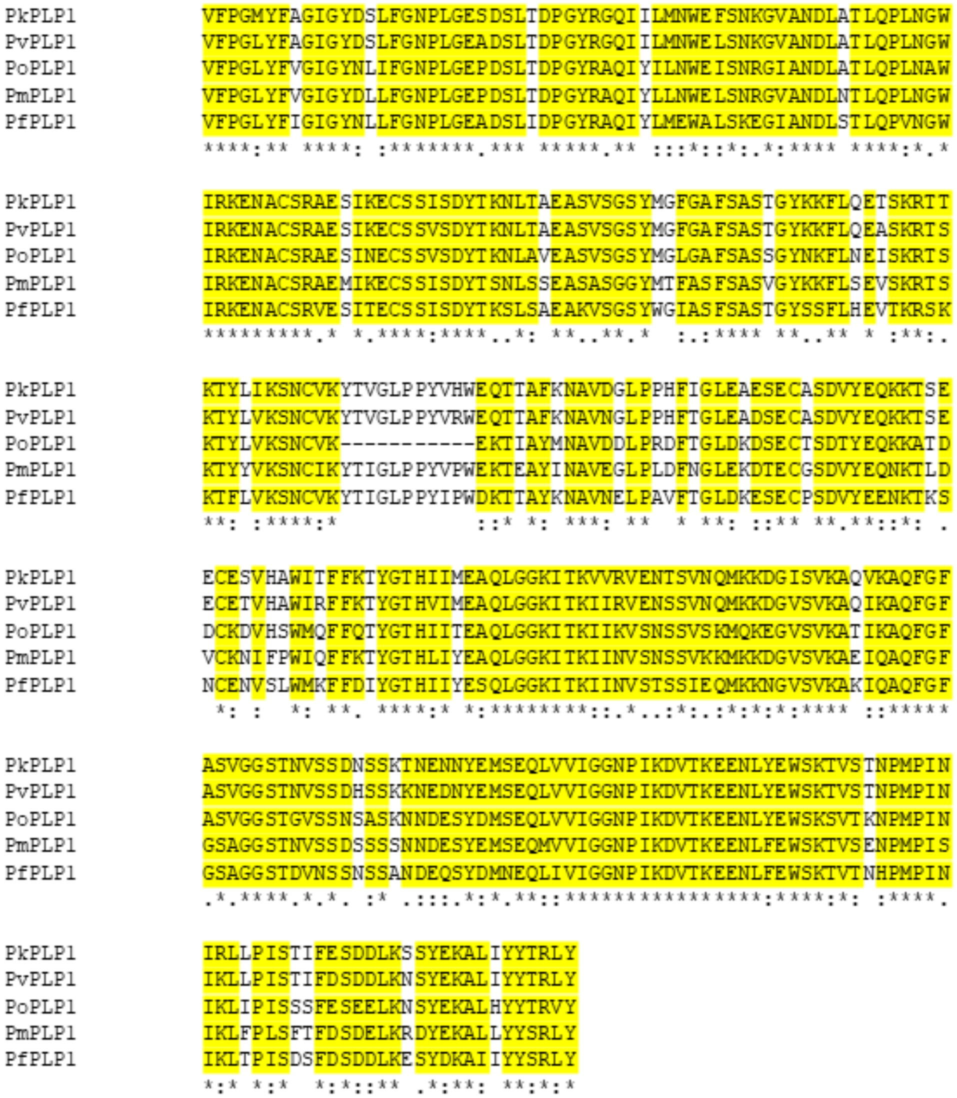

Supplementary Figure S3. Sequence conservation of MAC domains between different *Plasmodium spp.* The ClustalW alignment of MACPF domains of *Plasmodium spp.* PLPs [*Plamodium falciparum* (PfPLP1), *Plasmodium vivax* (PvPLP1), *Plasmodium ovale* (PoPLP1), *Plasmodium knowlesi* (PkPLP1) and *Plasmodium malariae* (PmPLP1)] is shown. Conserved residues are highlighted in yellow.

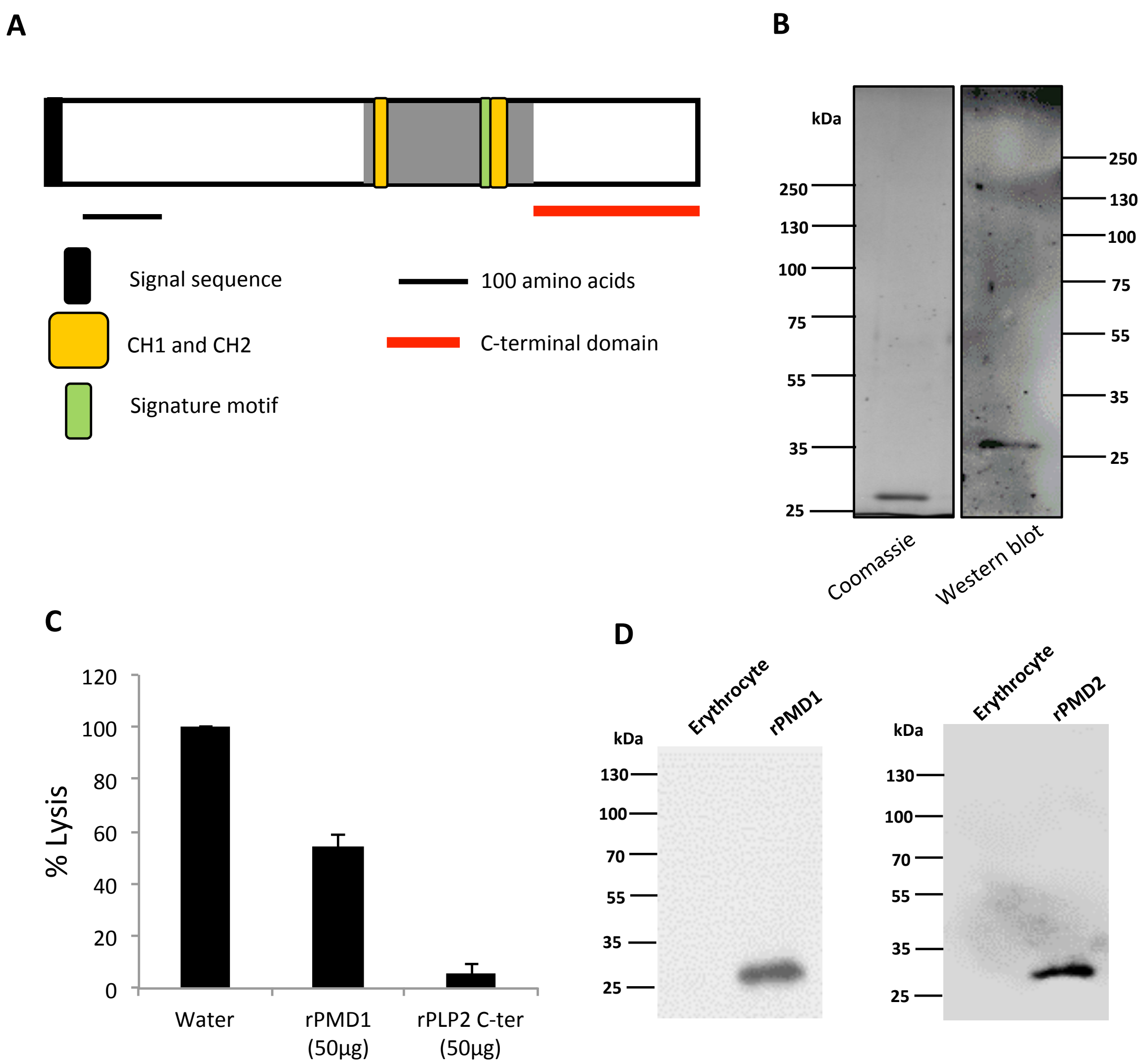

Supplementary Figure S4. Activity of rPMDs. (**A**) Domain architecture of PfPLP2. The signature motif (green box) and two transmembrane helical domains, CH1 and CH2 (yellow boxes) are depicted. Red line indicates C-terminal domain. Scale bar represents 50 aa. (**B**) Coomassie and Western blot of affinity-purified rPLP2 C-ter domain probed with the anti-his antibody. (**C**) The lysis of human erythrocytes was analyzed in the presence of 50µg each of rPMD1 or rPLP2 C-terminal. The graph indicates the percent lysis of human erythrocytes as compared with 100% hypotonic lysis in the presence of water. A non-lytic rPLP2 C-terminal histidine tagged protein was taken at the same concentration to show specific activity of rPMD1. (**D**) The erythrocytes were incubated with rPMD1 or rPMD2 and detected by Western blotting. Full blots from Figure 1F are represented.

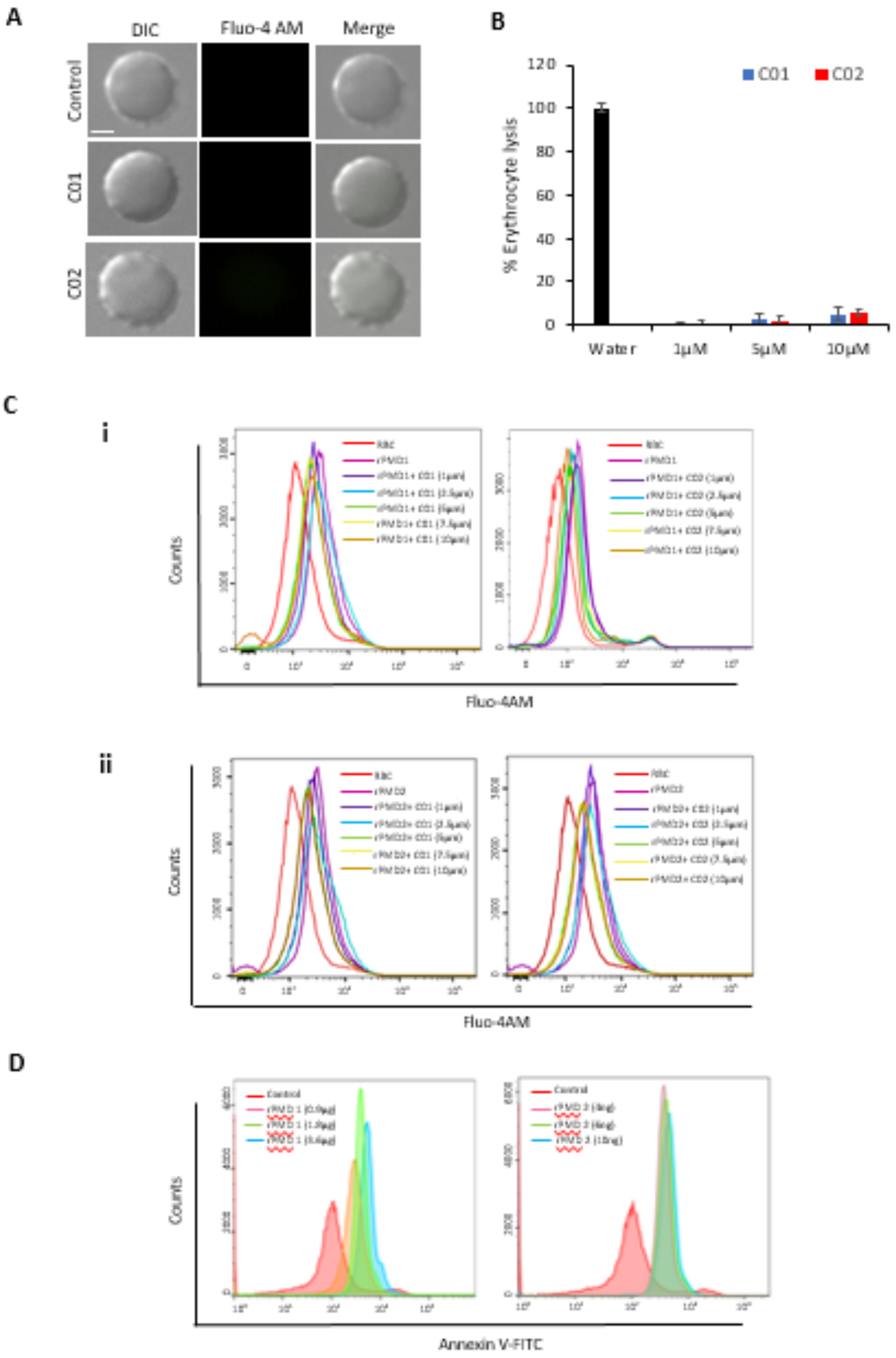

Supplementary Figure S5. PMI mediated inhibition of Calcium influx and Annexin positivity. (**A**) Fluo-4 AM stained erythrocytes were treated with PMIs for 1 h at 37°C and imaged for calcium increase. (**B**) Erythrocytes were treated with different concentrations of PMIs for 1 h at 37°C and the hemoglobin release was measured. The graph indicates the percent lysis of human erythrocytes as compared with 100% hypotonic lysis of erythrocytes in water. (**C**) The Fluo-4 AM loaded erythrocytes were treated with rPMD1 (i) and rPMD2 (ii) in the presence and absence of indicated concentration of PMIs and calcium increase was monitored by Flow cytometer. (**D**) The erythrocytes were treated with rPMD1 or rPMD2 and stained with Annexin V-FITC after 48 h. The annexin positive erythrocytes were quantitated using a flow cytometer.

A

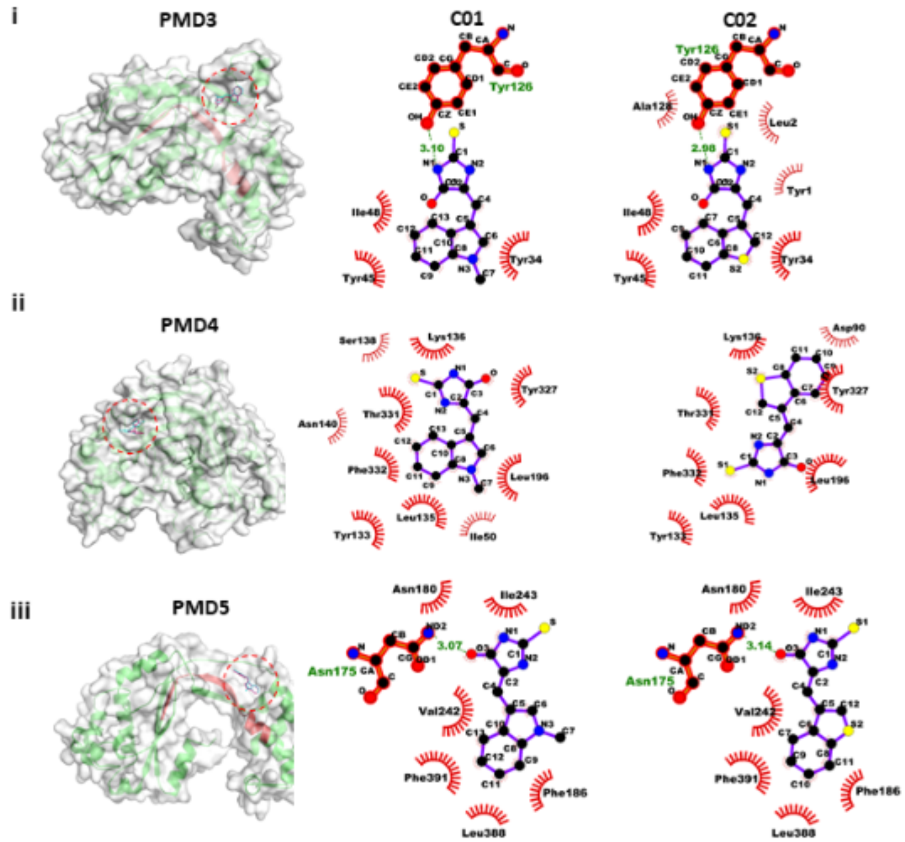

B

Binding Energy (kcal/mol)

	PMD1	PMD2	PMD3	PMD4	PMD5
**C01**	-6.0	-6.6	-4.9	-7.6	-6.8
**C02**	-6.0	-6.8	-6.7	-7.0	-6.6

C

```
PfPLP1   -SNCENVSLWMKFFDIYGTHIIYESQLGGKITKIINVSTSSIEQMKKNGVSVKAKIQAQF
PfPLP2   PECEENVNKWMLFFKLHGTHVAYEIYLGGKIIIKININKEEYNKMKENNINVKTFFNIYF
PfPLP3   EKCGKSVTAWMYFFKNFGTHVSTLLHLGGKITQQVKISKNDYKAMTESGLSISASVSAGF
PfPLP5   ENCLKTITPWISFFNMYGTHVISGVYYGGKIIHNLYFENNNLKKKEYKIRMYKSRLNPFS
PfPLP4   -HCSEGIKSWMKFFEKYGTHVVLSAHFGGMSFNTMEITKRKIEEIKIYKYKYSLWNNPYL
             : :. *: **. .***:      **     : . . . :         .   .
```

Supplementary Figure S6. Binding of C01 and C02 to the MACPF domains. (**A**) The Clustal W alignment of the sequences of PMD1-5 where PMIs are binding. Conserved residues are shown in red, blue and purple. (**B**) The surface images of docked complexes showing binding of C01 and C02 to the signature motif of PMD3, PMD4, and PMD5. The ligplot figures demonstrate the specifics of the atoms involved in the interaction. (**C**) The table summarizes the binding energies of PMI interaction to PMDs.

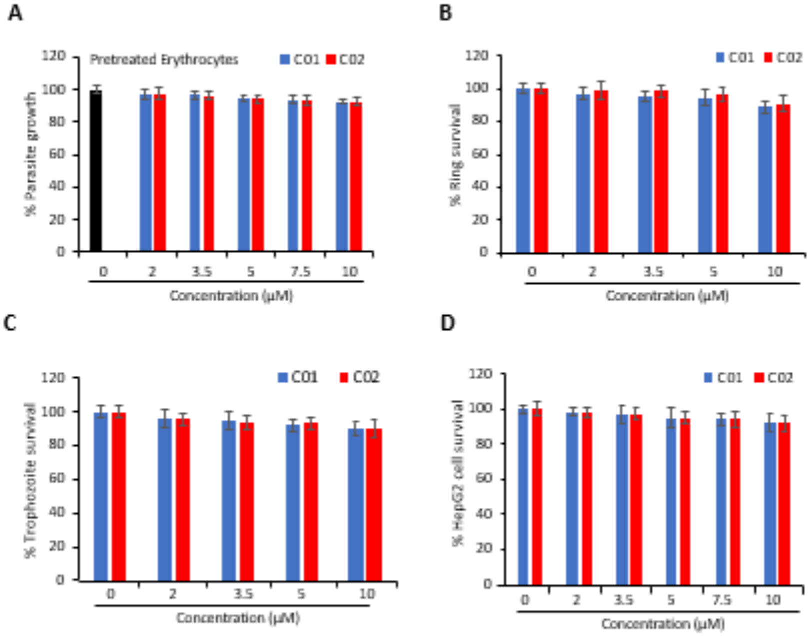

Supplementary Figure S7. Toxicity analysis of C01 and C02. (**A**) Erythrocytes were pre-treated with different concentrations of PMIs for 3 h and washed. Percoll purified schizonts were added and the parasite invasion was scored by counting after 8 h using Giemsa stained smears. (**B**) Ring stage parasites were treated with different concentrations of compounds for 6 h and washed, the inhibition was accessed by counting the Giemsa smears after one cycle of parasite growth. (**C**) Trophozoite stage parasites were treated with different concentrations of compounds for 6 h and the inhibition was accessed by counting the Giemsa smears after one cycle of parasite growth. (**D**) The HepG2 cells were treated with a higher concentration of PMIs and toxicity was assessed using MTT assay.
